# Yes-associated protein regulates autophagy to restore skin barrier function in atopic dermatitis

**DOI:** 10.3389/fimmu.2025.1681148

**Published:** 2025-11-19

**Authors:** Jinjing Jia, Xin Ma, Tongfei Wang, Siqi Ye, Fenggen Yan, Junfeng Liu, Hongyi Li, Dacan Chen, Xiumei Mo

**Affiliations:** 1 StateKey Laboratory of Traditional Chinese Medicine Syndrome/State Key Laboratory of Dampness Syndrome of Chinese/Department of Dermatology, the Second Affiliated Hospital of Guangzhou University of Chinese Medicine, Guangzhou, China; 2 The Second Clinical College of Guangzhou University of Chinese Medicine, Guangzhou, China

**Keywords:** atopic dermatitis, yes-associated protein, skin diseases, eczema, autophagy, inflammation

## Abstract

**Introduction:**

Atopic dermatitis (AD) is characterized by skin barrier dysfunction and inflammation.This study explored the relationship between Yes-associated protein (YAP) expression, autophagy, and skin barrier dysfunction in AD.

**Methods:**

Skin samples from AD patients and healthy controls were analyzed using mRNA-seq. Differential gene expression was visualized using ggplot2 and analyzed using GO and KEGG pathway analyses. An AD mouse model was created using house dust mite ointment, and HaCaT cells were treated to mimic AD inflammation. YAP expression was transfected with lentivirus *in vivo* and siRNA *in vitro*. Autophagy was induced with rapamycin.

**Results:**

YAP levels were reduced in AD patients and correlated with filaggrin (FLG). YAP overexpression in mice improved skin lesions, enhanced barrier function (increased FLG, involucrin, and loricrin), and promoted autophagy (increased LC3-II/I; decreased p62 and p-mTOR). Similar effects were observed in cells, with increased proliferation and reduced apoptosis. YAP knockdown reversed these effects, which were mitigated by rapamycin.

**Discussion:**

Reduced YAP expression in AD is linked to inflammation and barrier dysfunction. YAP may improve AD by enhancing autophagy, reducing inflammation, and restoring skin barrier function.

## 1 Introduction

Atopic dermatitis (AD), also known as atopic eczema, is a chronic, recurrent, and itchy skin disease that affects a wide range of people (1). The prevalence of AD is 20% in children and 10% in adults, with a gradually increasing trend (2). The exact pathogenesis of AD is not yet clear, but it is currently believed to be caused by skin barrier dysfunction and immune dysfunction due to the stimulation of various antigens and microbial products in the environment under a genetic background favoring the disease (3). Dysfunction of the skin barrier is a hallmark of AD and an initiating link in its pathogenesis. It makes the skin susceptible to the invasion of allergens and microorganisms, leading to immune-inflammatory disorders (4). Skin barrier dysfunction is accompanied by a series of changes in epidermal structural proteins such as filaggrin (FLG), involucrin (IVL), and loricin (LOR) expression defects, reduced lipid layer formation, decreased ceramide expression, and reduced tight junction structures (5).

Current treatments for AD are varied and depend on the individual clinical variability of the disease (2, 6). Basic therapy involves the treatment of barrier dysfunction through hydrating and lubricating topical treatments, alongside avoidance of symptom-provoking factors. Long-term management and control of flare-ups can involve topical anti-inflammatory treatment, such as corticosteroids and calcineurin inhibitors, but topical phosphodiesterase inhibitors may be an alternative option. UV irradiation can be used as an adjuvant therapy (6). Despite these treatments, many patients with severe AD reported such treatments to be ineffective and with many side effects (7). As a first-line topical medication, glucocorticoids have strong anti-inflammatory effects, but long-term use can worsen the damage to the skin barrier. Many patients endure recurrent pain, drug toxicity, and side effects (8). For severe cases, biological agents or immunosuppressants can be considered. However, the efficacy of biological agents in AD is still limited compared with other diseases, such as psoriasis, and its recurrence cannot be avoided (9). Hence, it is very important to study more effective drugs that can simultaneously regulate the immune balance disorder of AD and restore the skin barrier. Fortunately, multiple agents targeting specific disease pathways are becoming available, presenting a more optimistic outlook for patients (7). Therefore, it is important to fully understand the mechanisms involved in developing AD to identify new therapeutic targets.

Recent studies have shown that autophagy levels are reduced in AD. Autophagy also plays an important role in the pathogenesis of AD by regulating inflammatory responses, keratinocyte differentiation, and host defense mechanisms against invading pathogens (10) such as *Staphylococcus aureus* (11), which widely colonizes the skin of patients with AD (12). Inhibiting the expression of autophagy proteins can inhibit the expression of FLG, indicating a link between autophagy and reduced epidermal barrier function (13). Therefore, regulating autophagy activity may be a pharmacological option for treating AD. Topical autophagy enhancers can alleviate the severity of skin lesions, transepidermal water loss (TEWL), and itching in AD patients (14). However, much remains to be discovered about the mechanisms involved in autophagy. Understanding the details may reveal related therapeutic targets for AD that have yet to be investigated.

The Hippo signaling pathway is vital for regulating the development, homeostasis, and regeneration of the dermis and epidermis, and it is important in protecting the skin’s barrier function (15). Yes-associated protein (YAP) is a key downstream effector of the Hippo signaling pathway (15). YAP is upregulated in various malignant tumors and may regulate cell proliferation and apoptosis, tissue and organ growth and size, epithelial-mesenchymal transition, and intercellular contact inhibition and participate in stem cell self-renewal (16). YAP plays an important role in regulating the proliferation and differentiation of skin keratinocytes, maintaining the three-dimensional structure of the skin, and sustaining cellular autophagy (17). Previous research by the authors found a decrease in YAP levels in AD skin lesions (18), suggesting that it may be involved in the core pathogenesis of AD skin barrier dysfunction. It leads to hypothesize that downregulated YAP in AD reduces autophagy, influencing skin barrier function, and that increasing YAP may help alleviate AD. Therefore, to investigate this hypothesis, this study explored the relationship between YAP expression levels, autophagy levels, and skin barrier dysfunction in AD at the clinical, animal, and cellular levels. The results may suggest new therapeutic targets for AD.

## 2 Materials and methods

### 2.1 Patients and samples

Skin tissue samples were collected from patients with AD who visited the Dermatology Department of Guangdong Provincial Hospital of Traditional Chinese Medicine between November 2020 and July 2023. Normal skin tissue was obtained from healthy control subjects who had cosmetic surgery in outpatient clinics at the hospital during the same period. All patients with AD met the diagnostic criteria of Hanifin-Rajka (19) and Williams (20). Patients who have received systemic treatment within the past four weeks or who have any uncontrolled systemic disease were excluded. Local anesthetics were used and then biopsies with a length of 5mm were performed. The score for the severity of AD (SCORAD) (21) was recorded for patients with AD prior to biopsy. The study was approved by the Ethics Committee of Guangdong Provincial Hospital of Traditional Chinese Medicine (approval number YF2022-315-01). All participants provided written informed consent (signed by their parents or guardians for patients under 18).

### 2.2 Histopathology and immunohistochemistry

All skin tissue specimens were fixed in 4% paraformaldehyde and embedded in paraffin for histopathological and immunohistochemical (IHC) staining. Hematoxylin & eosin (HE) and IHC staining followed standard operating procedures (22, 23), and 4 μm thick sections were prepared with slicer (RM2016, Leica, Germany). Deparaffinization was performed using xylene, and then antigen retrieval and endogenous peroxidase blocking. After blocking with blocking buffer for 1 hour, incubation with the primary antibody at 4 °C overnight followed, and the results were observed under a microscope (BX53, OLYPUS, Japan). The staining intensity (yellow, brownish yellow, or brown granules) was evaluated at four levels: 0 points, negative; 1 point, weakly positive; 2 points, positive; 3 points, strongly positive. The IHC score was calculated using strong positive × 3+positive × 2+weak positive × 1 (24). Three fields of view at ×40 magnification were randomly selected for each slide to obtain the average value. The primary antibodies used were against YAP (1:400, Cell Signaling Technology, #14074) and FLG (1:200, Affinity Biosciences, DF13653, for human; 1:100, Gene Tex, GTX23137, for mice).

### 2.3 Establishment of the AD mouse model

Nishiki-nezumi Cinnamon/Nagoya (NC/Nga) mice, aged 6–8 weeks, were allocated into nine groups with six mice in each group (3 males and 3 females). In the experimental groups, the back and bilateral ears had hair removal cream (Veet, UK) applied, and then after 24 hours, 200 μL of 4% SDS was applied to the same area. Next, 100 mg of house dust mite (HDM) (*Dermatophagoides farinae*) ointment was applied after 2 hours, and this was applied to the same area repeated twice a week for 3 weeks (25, 26). In the control group, an equal volume of matrix Vaseline was applied.

The YAP overexpression groups (YAP and AD+YAP) were injected with YAP overexpression lentivirus at the lesion site, while the YAP silencing groups (sh-YAP, AD+sh-YAP, and AD+sh-YAP+RPM) were injected with YAP shRNA lentivirus (**Supplementary Table S1**) at the lesion site (27, 28). The lentiviral was diluted with sterile physiological saline and concentrated to a titer of 2×10^7^ TU/mL, with an injection volume of 100 μL. The lentiviral encapsulation system comprised transfer, packaging, and envelope plasmids. The rapamycin group (AD+sh-YAP+RPM) was treated with 0.2% rapamycin ointment twice a week, prepared from rapamycin powder (Maclin MACKLIN) and Vaseline (29).

The scoring method for dermatitis in mice includes four indicators: (1) erythema/hemorrhage, (2) scarring/dryness, (3) edema, (4) excoriation/erosion (30). The severity of each indicator is calculated on a scale of 0-3, with 0=none, 1=mild, 2=moderate, and 3=severe. The total score is the dermatitis score. TEWL and stratum corneum hydration (SCH) were measured using a skin detector (GPskin Barrier Light). The thickness of skin lesions and the degree of eosinophil infiltration were detected using histopathological HE examination results. At least 3 high-power slides at a magnification of ×200 were counted for each mouse to obtain the average value.

### 2.4 Establishment of the AD-like inflammatory cell model

The human immortalized keratinocyte cell line HaCaT was cultured routinely in Dulbecco’s modified Eagle medium (DMEM) (Gibco) and 10% fetal bovine serum (11011-8611, Tianhang) at 37 °C and 5% CO_2_. The Lipofectamine 3000 transfection reagent (L3000015, Invitrogen) was diluted with an equal volume of opti-MEM medium to deliver the plasmid, siRNA or vector into cells for 48 hours. The YAP overexpression group was transfected with the YAP overexpression plasmid, while the YAP knockdown group was transfected with siRNA (**Supplementary Table S1**). A mixture of 10 ng/mL of interferon-gamma (IFN-γ) (SinoBiological) and 10 ng/mL of tumor necrosis factor-alpha (TNF-α) (SinoBiological) was used to treat HaCaT cells in the logarithmic growth phase for 24 hours to create an AD-like inflammatory cell model (31, 32). The rapamycin group was treated with 50 nM rapamycin (33). Furthermore, levels of mTOR were measured in AD-like inflammatory cell model exposed to YAP overexpression or knockdown at different time points (0, 6, 12, 24 hours).

### 2.5 Detection of protein expression by western blot

Total protein was extracted from skin lesions or cells, and its concentration was determined using the bicinchoninic acid (BCA) method. Western blotting was performed through sodium dodecyl-sulfate polyacrylamide gel electrophoresis (SDS-PAGE) and then transferred to PVDF membranes. After blocking with block buffer (wanleibio, WLA066), antibody incubation and visualization to detect protein expression. The primary antibodies used were YAP (1:1000, Cell Signaling Technology, #14074), p-YAPS127 (1:1000, Cell Signaling Technology, #13008), FLG (1:1000, Gene Tex, GTX23137), IVL (1:400, bioss, bs-23062R), LOR (1:1000, novus, NBP1-33610), LC3A/B (1:1000, Cell Signaling Technology, #12741S), p62 (1:1000, Cell Signaling Technology, #39749S), mTOR (1:500, wanleibio, WL02477), p-mTOR (1:500, wanleibio, WL03694), β-actin (1:1000, wanleibio, WL01372), Beclin-1 (1:1000, wanleibio, WL02508), ATG5 (1:1000, wanleibio, WL02411), and ATG7 (1:500, wanleibio, WL02793). The horseradish peroxidase-marked second antibodies were incubated at 37 °C for 45 minutes (1:5000, wanleibio, WLA023 and WLA024, for mice and cells; 1:20000, 4050-05, Southern Biotech, for human).

### 2.6 Detecting cytokine levels in serum by ELISA

Venous blood was collected from each mouse, and the serum was obtained. Enzyme-linked immunosorbent assays (ELISAs) were used to detect the levels of IgE, IL-4, and IL-17A cytokines in the serum with the following kits: Mouse IgE ELISA Detection Kit (EIAab, E0545m), Mouse IL-4 ELISA Detection Kit (EIAab, E0077m), and Mouse IL-17A ELISA Detection Kit (EIAab, E0063m). Detecting cytokine levels in serum according to the manufacturer’s protocol.

### 2.7 Immunofluorescence staining

Cell slide coverslips or tissue sections were incubated with a concentration of 0.1% TritonX-100 at room temperature for 30 minutes. Wash three times with phosphate-buffered saline (PBS) buffer, for 5 minutes each time. After washing, the primary antibodies were added and incubated overnight at 4 °C. The fluorescence-labeled secondary antibody was added and incubated at room temperature for 60 minutes. After washing, 4’6-diamidino-2-phenylindole (DAPI) was added to counterstain the nucleus, and an anti-fluorescence quencher (S2100, Solarbio) was used to seal the slide before observation under a fluorescence microscope (BX53, OLYMPUS). The fluorophores include Cy3 secondary antibody (red), FITC secondary antibody (green), and DAPI (blue). Histology was performed on samples from the mice. Sections were stained with HE to show the tissue structure and with toluidine blue dye (Solarbio, No. G3662) to investigate mast cell density according to standard procedures. Immunofluorescence staining was used to detect the infiltration of CD4+ interleukin (IL)-17A+double positive cells (i.e., Th17 cells) in the dorsal skin lesions of mice in each group. Immunofluorescence staining was used to detect the expression of autophagy marker light chain 3 (LC3) (Cell Signaling Technology, 43566S) and YAP (Santa Cruz, sc-101199) in the mice or cells of each group.

### 2.8 mRNA sequencing

Three patient and control samples underwent analysis of the mRNA expression levels. Total RNA was extracted from the samples, followed by mRNA enrichment and capture using Oligo (dT) magnetic beads, cDNA obtainment and purification. Subsequently, the purified cDNA was subjected to adapter ligation, PCR enrichment, and library sequencing. The alignment of reads to a reference genome is performed using the HISAT2 software (v2.2.1). Differentially expressed genes (DEGs) was performed using DESeq2 (v1.28.0), with the criteria set at false discovery rate (FDR) <0.05 and log2 (fold change) > 1 to indicate significant differential expression. The volcano plot was drawn using the ggplot2 package in R (version 4.2.0; https://www.r-project.org/). During analysis, for DEGs annotated by Gene Ontology (GO) terms, calculations were made for each term’s gene list and the number of genes, followed by computation of P-values through clusterProfiler to identify the main biological functions of the DEGs. The R package clusterProfiler was employed to calculate significantly enriched pathways of differential genes, identifying the pathways significantly enriched among differentially expressed genes.

### 2.9 Transmission electron microscopy

After undergoing routine steps such as fixation in fixative (G1102, Servicebio, China) at 4 °C for 2 hours, subsequent embedding, dehydration, polymerization, and ultra-thin sectioning (approximately 80 nm) with copper grids and then staining, the formation of autophagosomes (phagosomes, autophagosomes, and autolysosomes) in each group was observed under transmission electron microscopy (Hitachi, Japan).

### 2.10 Cell proliferation assay

The 3-(4,5-dimethylthiazol 2-yl)-2,5-diphenyltetrazolium bromide (MTT) assay was used to detect cell proliferation. Cells in logarithmic growth were seeded into a 96-well plate, with 5 × 10^3^ cells per well and five replicates per group. After treating the cells described above for 24 hours, 20 μL of MTT (wanleibio) was added to each well. After incubating in the dark for 4 hours, the supernatant was discarded, and 150 μL of formazan (wanleibio) solution was added to each well. The plates were shaken until all formazan was dissolved, and the absorbance values of each well were measured using a microplate reader (BIOTEK, USA) at a wavelength of 570 nm.

### 2.11 Cell apoptosis assay

Cells were seeded into six-well plates, with three replicates per group, and treated according to their experimental groups. The cells were harvested according to the apoptosis detection kit (wanleibio), and 500 μL of binding buffer was used to gently resuspend the cells. Then, 5 μL of annexin V-FITC was added and mixed well, followed by 10 μL of propidium iodide staining solution and mixing well. The cells were incubated at room temperature in the dark for 15 minutes, and then flow cytometry was performed with a NovoCyte Flow Cytometer (Agilent Technologies, Inc., USA).

### 2.12 Statistical methods

SPSS 19.0 statistical software (IBM Corp., USA) was used for analysis. The data were expressed as mean ± standard deviation. The means were compared between two groups using Student’s t-test, while comparisons among three or more groups were conducted using analysis of variance, and pairwise comparisons between groups were conducted using the least significant difference (LSD) method or Dunnett T3 method was used for variables with uneven variances. Correlation analysis of FLG and YAP expression levels was conducted using the Pearson method. *P* < 0.05 indicated a statistically significant difference.

## 3 Results

### 3.1 YAP decreases in the skin lesions of patients with AD and is positively correlated with the expression of FLG

**Figure 1:**
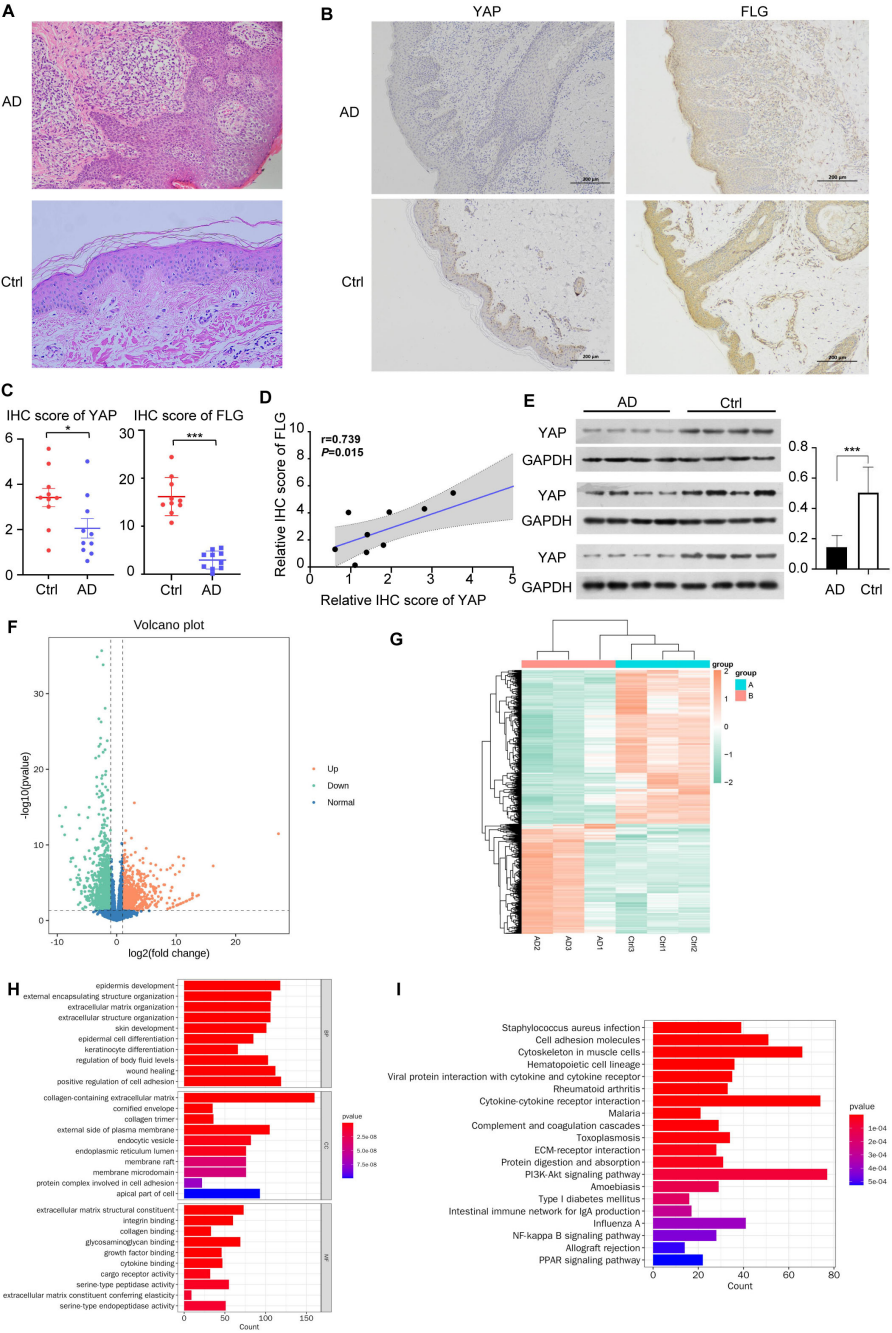
YAP expression and mRNA sequencing in clinical samples. **(A)** Hematoxylin & eosin (HE) staining of AD skin lesions and healthy controls (×200). n = 10. **(B)** Immunohistochemical results of YAP and Filaggrin (FLG) in AD skin lesions and healthy controls (×100). n = 10. **(C)** YAP and FLG protein expression and quantitative statistics of the skin samples in AD patients and healthy controls. n = 10. **(D)** Pearson correlation analysis between YAP and FLG expression in patients with AD. **(E)** Western blots of YAP. n = 12. **(F)** Volcano plot showing the differential expression of mRNAs between the AD samples (n = 3) and controls (n = 3). **(G)** Clustering heatmap analysis of differential expression genes (DEGs). **(H)** Analysis of biological processes in which the upregulated/downregulated genes are involved. **(I)** Analysis of the pathways in which the upregulated/downregulated genes are involved. AD: atopic dermatitis patient samples; Ctrl: healthy control samples. *P < 0.05, ***P < 0.001 v. s. AD.

The demographics of the study participants are shown in **Table 1**, samples were collected from 12 patients with AD (seven males and five females, aged 14–80 years) and 12 healthy controls (seven males and five females, aged 13–74 years) to analyze protein levels. Among them, 10 patients with AD (six males and four females, aged 14–80 years) and 10 healthy controls (six males and four females, aged 13–74 years) also provided samples for immunohistochemistry, and three patients with AD and three healthy controls also provided samples for mRNA sequencing analysis. The AD and control participants were matched in terms of sex and had similar ages (38.1 ± 18.1 years in the AD group versus 35.8 ± 17.3 in the control group). The mean score for SCORAD of the patients with AD was 58.0. The characteristics of the AD lesions in the patients’ skin were explored. HE staining showed that compared with healthy skin tissue, AD skin lesions had thickened epidermis, obvious infiltration of inflammatory cells, sponge edema, and basal liquefaction under the microscope (**Figure 1A**).

**Table 1:** Clinical demographics of the expression of YAP.

Characteristics	AD (n = 12)	HC (n = 12)
Age (y), mean ± SD	38.1 ± 18.1	35.8 ± 17.3
Gender, no. (%)		
Female	5	5
Male	7	7
Clinical severity		
IGA, mean ± SD	3.7 ± 0.7	N/A
SCORAD, mean ± SD	57.9 ± 15.0	N/A
EASI, mean ± SD	20.8 ± 9.8	N/A
Total IgE (IU/mL), mean ± SD, range	1186.7 ± 866.8 (142.6, 2500.0)	N/A
Blood EOS (10*9/L), mean ± SD, range	0.8 ± 0.6 (0.17, 2.18)	N/A

AD, atopic dermatitis; HC, Health control; IGA, Investigator Global Assessment; SCORAD, SCORing Atopic Dermatitis; EASI, Eczema Area and Severity Index; EOS, Eosinophil; IgE, Immunoglobulin E; SD, standard deviation.

IHC confirmed that the YAP and FLG protein expression levels in the AD group were lower than in the control group (**Figure 1B**).

Western blotting confirmed that compared with the healthy control group, YAP and FLG expression in the skin lesions of the 12 patients with AD was generally lower (**Figures 1C, E**). Correlation analysis results showed a significant positive correlation between FLG and YAP protein expression in the AD disease group (correlation coefficient=0.739, and *P* = 0.015) (**Figure 1D**). There was no significant correlation between FLG and YAP expression levels in the healthy control group (correlation coefficient=0.053, *P*>0.05). Transcriptome analysis showed that in clinical AD samples compared to control samples, 1074 mRNAs were upregulated while 1502 mRNAs were downregulated (**Figure 1F**). The clustering analysis of DEGs revealed significant differences between each group (**Figure 1G**). Analysis of the processes these upregulated/downregulated mRNAs might be involved in suggested many processes related to skin barrier damage, such as keratinocyte differentiation, epidermal cell differentiation, and cornified envelope (**Figure 1H**). Many pathways were potentially involved, including PI3K/AKT signaling, *Staphylococcus aureus* infection, and cytokine-cytokine interaction (**Figure 1I**). Previous studies have confirmed that the PI3K/AKT/mTOR pathway is a classic inhibitory pathway for autophagy, while YAP can inhibit the PI3K/AKT/mTOR signaling pathway (34). This suggests that YAP may play a significant role in regulating autophagy through the mTOR pathway in the pathogenesis of AD.

### 3.2 The expression of YAP can affect the severity of skin lesions in AD mice

**Figure 2:**
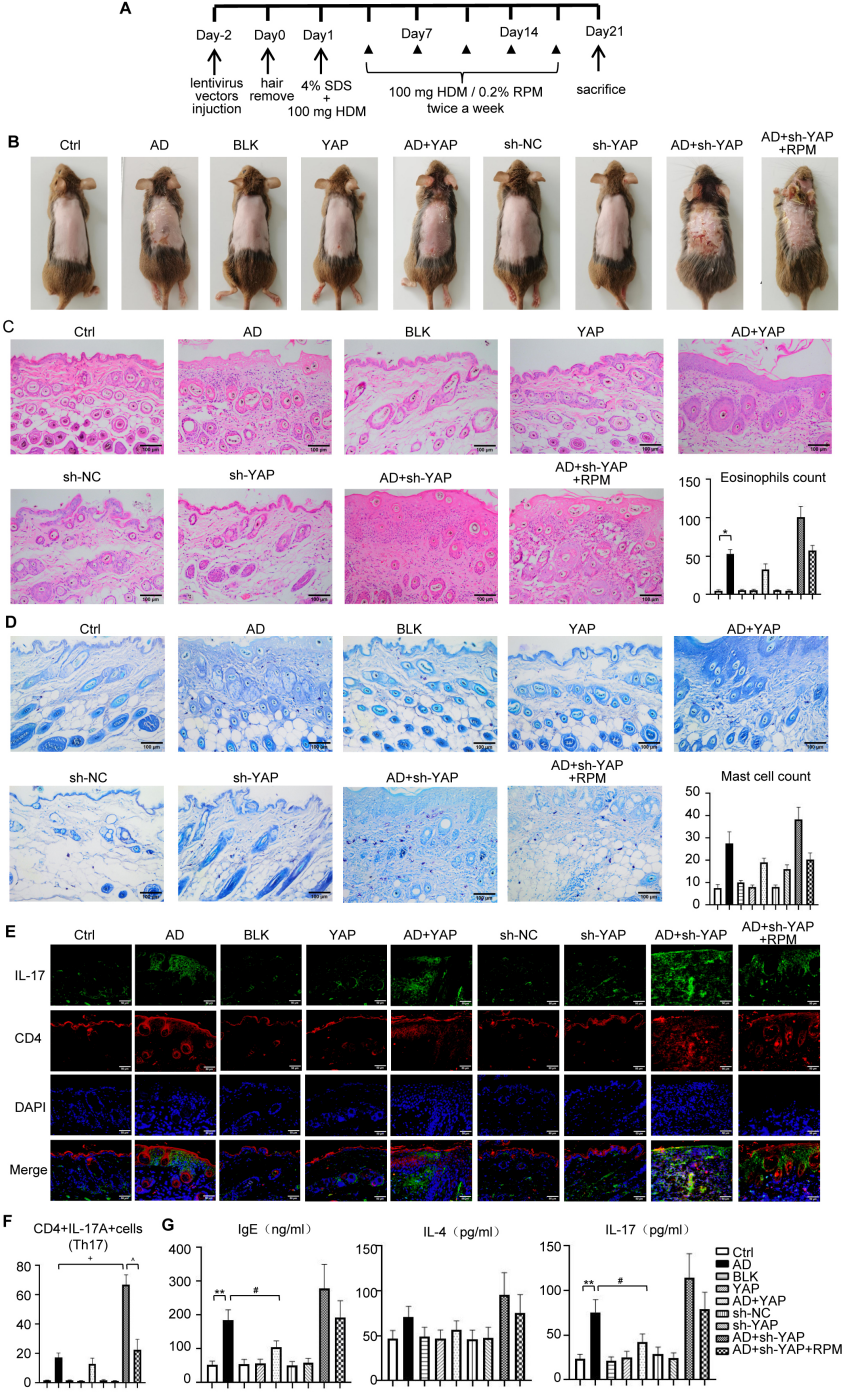
The effect of YAP expression on immune balance disorder in AD mice. **(A)** House dust mite (HDM)-induced atopic dermatitis (AD) mouse modeling scheme. **(B)** General pictures of the mice. **(C)** HE staining for histopathological features and eosinophils count (×200), bar length = 100 μm. **(D)** Toluidine blue staining to identify mast cells (×200), bar length = 100 μm. **(E)** CD4+IL-17A+cells (Th17) counts in immunofluorescence (×400), bar length = 50 μm. **(F)** Quantification of immunofluorescence results of CD4+IL-17A+cells (Th17) (×400). IL-17: green. CD4: red. **(G)** IgE, IL-4, and IL-17 expression in mouse serum from ELISA. Ctrl, control mice; AD, atopic dermatitis model mice; BLK, mice injected with empty vector lentivirus; YAP, mice injected with YAP overexpression lentivirus; AD+YAP, atopic dermatitis model mice injected with YAP overexpression lentivirus; sh-NC, mice injected with shRNA-NC lentivirus; sh-YAP, mice injected with YAP shRNA lentivirus; AD+sh-YAP, atopic dermatitis model mice injected with YAP shRNA lentivirus; AD+sh-YAP+RPM, AD+sh-YAP group with topical 0.2% rapamycin ointment. *P < 0.05, **P < 0.01 Ctrl v.s. AD; #*P* < 0.05 AD+YAP v.s. AD; +*P* < 0.05 AD+sh-YAP v.s. AD; ^*P* < 0.05 AD+sh-YAP+RPM v.s. AD+sh-YAP.

The AD model was successfully established in mice according to the schedule shown in **Figure 2A**, with lesions similar to those of patients with AD in both clinical and pathological aspects. The dermatitis score, ear thickness, TEWL, and SCH were similar between mice of different sexes (**Supplementary Tables S2**-**S4**). Compared with the AD group, the AD+YAP group had slightly but not significantly milder skin lesions, lower dermatitis scores, decreased ear swelling, and skin lesion thickness, while the AD+sh-YAP group had more severe skin lesions, increased dermatitis scores, increased degree of ear swelling, and an increasing trend in skin lesion thickness. The application of rapamycin (to induce autophagy) in the AD+sh-YAP+RPM group slowed down the progression of the mouse skin lesions and relieved ear swelling, but it did not recover completely (**Figure 2B**, **Table 2**). Overexpression of YAP alone can also cause ear swelling and increased skin thickness (*P* < 0.05 compared to the Ctrl group and the BLK group), while knocking down YAP alone showed no changes in ear thickness (*P*>0.05 compared to the Ctrl group and the sh-NC group). So, these results suggest that YAP expression decreases the severity of AD, while YAP knockdown increases its severity.

**Table 2:** Dermatitis score, ear thickness, and skin lesion thickness of each group of mice.

Group	Dermatitis score	Ear thickness(mm)	Skin lesion thickness
Ctrl	0	0.15 ± 0.01	24.17 ± 3.56
AD	3.67 ± 1.03***	0.19 ± 0.02***	127.62 ± 13.23*
BLK	0	0.15 ± 0.02	22.64 ± 3.72
YAP	0	0.17 ± 0.01	19.95 ± 2.56
AD+YAP	2.00 ± 0.89^##^	0.18 ± 0.02	104.97 ± 12.25
sh-NC	0	0.15 ± 0.01	22.20 ± 2.36
sh-YAP	0	0.16 ± 0.01	20.39 ± 2.07
AD+sh-YAP	6.50 ± 1.38^+++^	0.27 ± 0.02^+++^	256.01 ± 28.13
AD+sh-YAP+RPM	4.33 ± 1.51^^^	0.23 ± 0.01^^^	156.29 ± 19.78

***P < 0.001 Ctrl v.s. AD; ##*P* < 0.01 AD+YAP v.s. AD; +++*P* < 0.001 AD+sh-YAP v.s. AD; ^^^*P* < 0.001 AD+sh-YAP+RPM v.s. AD+sh-YAP.

### 3.3 The expression levels of YAP modulate inflammatory cell infiltration and cytokine expression in AD mice

The mechanisms involved in the role of YAP in AD were next investigated. Compared with the AD group, the AD+YAP group showed decreased infiltration of eosinophils and Th17 cells, while the AD+sh-YAP group showed increased infiltration of eosinophils and Th17 cells. The AD+sh-YAP+RPM group results suggested autophagy partially reduced the infiltration of eosinophils, Th17 cells, and mast cells (**Figures 2C-F**). Similar results were found for the expression of IgE, IL-4, and IL-17A although the difference was only statistically significant for IgE and IL-17 between the control and AD groups and the AD and AD+YAP groups (**Figure 2G**).

### 3.4 YAP expression in the epidermis of AD mice correlates with skin barrier function and modulates autophagy

**Figure 3:**
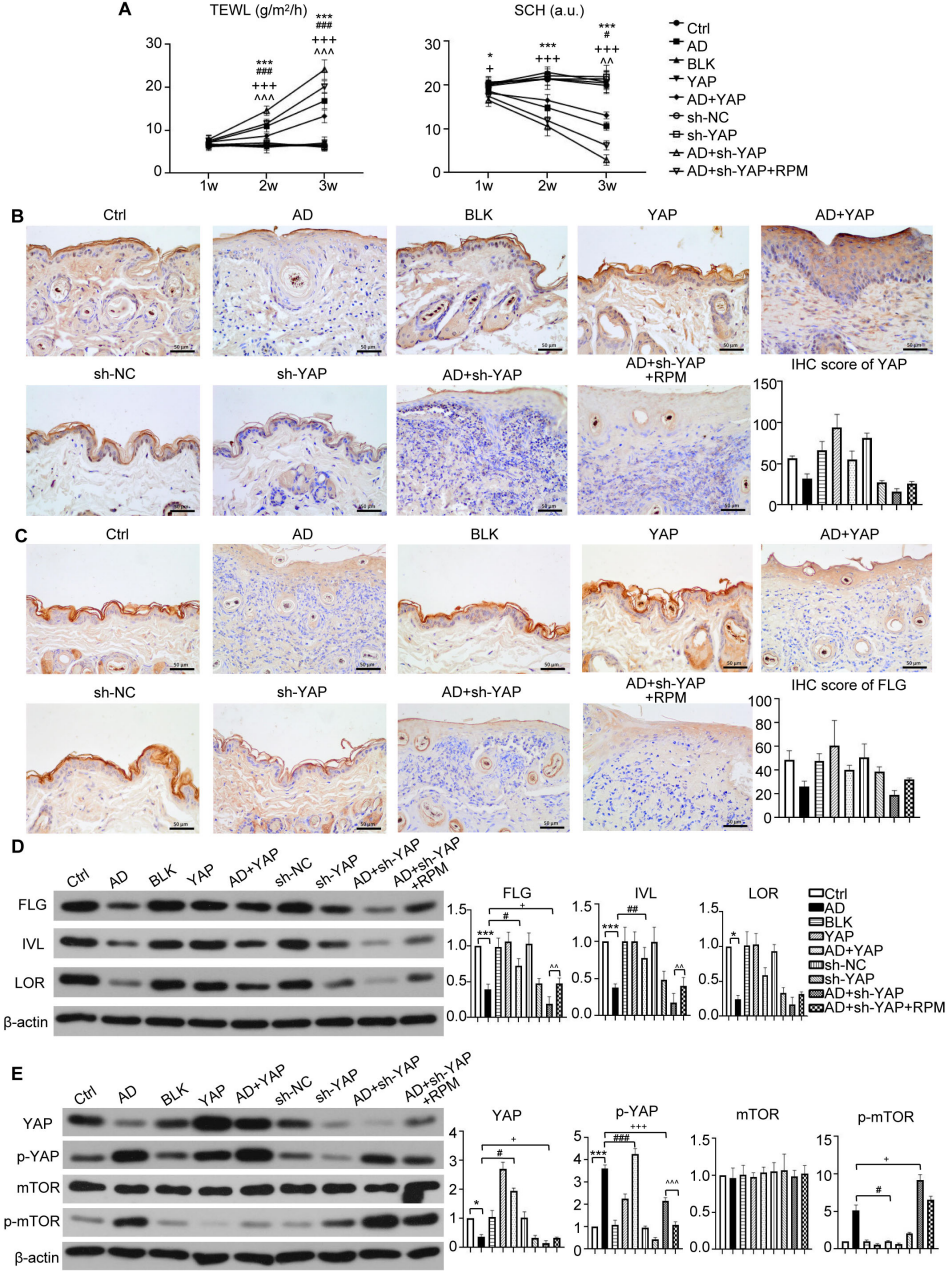
The effect of YAP expression on skin barrier in AD mice. **(A)** Trans epidermal water loss (TEWL) and stratum corneum hydration (SCH) of each group. **(B)** YAP protein expression from immunohistochemistry with the quantification results shown in the graphs (×400), bar length = 50 μm. n = 3. **(C)** Filaggrin (FLG) protein expression from immunohistochemistry with the quantification results shown in the graphs (×400), bar length = 50 μm. n = 3. **(D)** FLG, involucrin (IVL), and loricrin (LOR) protein expression from western blot with the quantification results shown in the graphs. **(E)** YAP, p-YAP, mTOR, and p-mTOR protein expression from western blot with the quantification results shown in the graphs. Ctrl, control mice; AD, atopic dermatitis model mice; BLK, mice injected with empty vector lentivirus; YAP, mice injected with YAP overexpression lentivirus; AD+YAP, atopic dermatitis model mice injected with YAP overexpression lentivirus; sh-NC, mice injected with shRNA-NC lentivirus; sh-YAP, mice injected with YAP shRNA lentivirus; AD+sh-YAP, atopic dermatitis model mice injected with YAP shRNA lentivirus; AD+sh-YAP+RPM, AD+sh-YAP group with topical 0.2% rapamycin ointment. *P < 0.05, ***P < 0.001 Ctrl v.s. AD; #*P* < 0.05, ##*P* < 0.01, ###*P* < 0.001 AD+YAP v.s. AD; +*P* < 0.05, +++*P* < 0.001 AD+sh-YAP v.s. AD; ^^*P* < 0.01, ^^^*P* < 0.001 AD+sh-YAP+RPM v.s. AD+sh-YAP.

TEWL exhibited a negative correlation with skin barrier function, while SCH displayed a positive correlation. In the YAP overexpression groups, the AD+YAP group showed decreased TEWL and improved SCH, indicating enhanced skin barrier function in comparison to the AD group. Conversely, YAP knockdown produced the opposite effects, the AD+sh-YAP group showed increased TEWL and decreased SCH, suggesting the worst skin barrier function, while the AD+sh-YAP+RPM group showed an improvement in these indicators (**Figure 3A**). IHC revealed an increasing trend of the expression of YAP and FLG protein in YAP overexpression group and a decrease in YAP knockdown group, consistent with the trend demonstrated in human samples (**Figures 3B, C**). FLG, IVL, and LOR protein levels increased with YAP overexpression and decreased with YAP knockdown. Rapamycin also increased the expression of FLG, IVL, and LOR in mouse skin lesions (**Figure 3D**). These results suggest that the expression of YAP may be significantly correlated with barrier dysfunction in AD.

**Figure 4:**
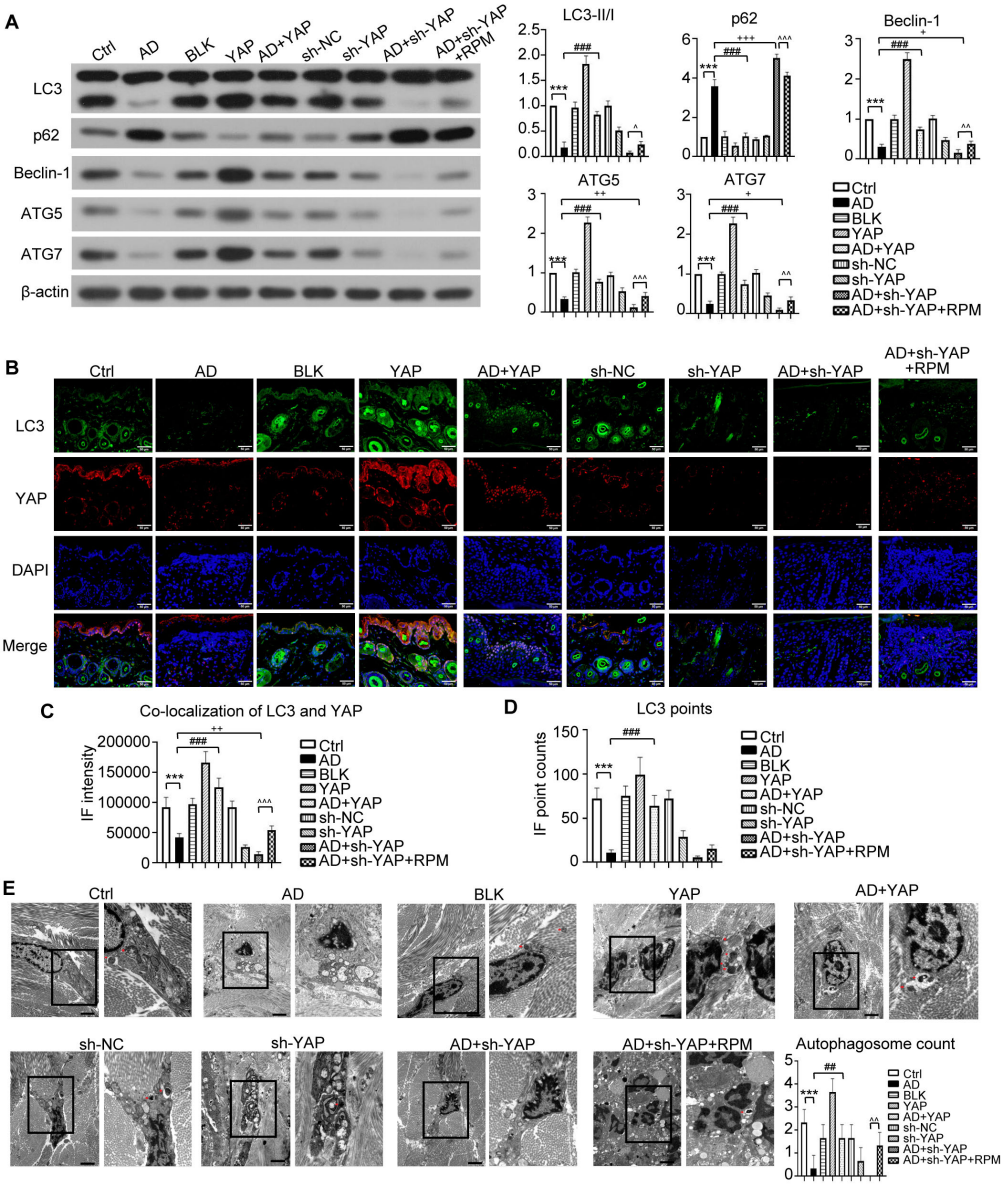
The effect of YAP expression on autophagy in AD mice. **(A)** Beclin-1, ATG5, ATG7, LC3, and p62 protein expression from western blot with the quantification results shown in the graphs. **(B)** LC3 positive cells (×600) and LC3-YAP co-localization (×400) from immunofluorescence, bar length = 50 μm. LC3: green. YAP: red. **(C, D)** Graphs showing co-localization of LC3 and YAP, and the LC3 points. **(E)** Electron microscopy results and autophagosome count (left: ×10,000; right: ×20,000; bar length = 2 μm). Ctrl, control mice; AD, atopic dermatitis model mice; BLK, mice injected with empty vector lentivirus; YAP, mice injected with YAP overexpression lentivirus; AD+YAP, atopic dermatitis model mice injected with YAP overexpression lentivirus; sh-NC, mice injected with shRNA-NC lentivirus; sh-YAP, mice injected with YAP shRNA lentivirus; AD+sh-YAP, atopic dermatitis model mice injected with YAP shRNA lentivirus; AD+sh-YAP+RPM, AD+sh-YAP group with topical 0.2% rapamycin ointment. ***P < 0.001 Ctrl v.s. AD; ##*P* < 0.01, ###*P* < 0.001 AD+YAP v.s. AD; +*P* < 0.05, ++*P* < 0.01, +++*P* < 0.001 AD+sh-YAP v.s. AD; ^^*P* < 0.01, ^^^*P* < 0.001 AD+sh-YAP+RPM v.s. AD+sh-YAP.

Since mTOR represents a well-established autophagy inhibitory pathway, we observed that while total mTOR levels remained constant, phosphorylated mTOR (p-mTOR) decreased following YAP overexpression and increased after YAP knockdown. Notably, rapamycin treatment induced a modest reduction in p-mTOR levels (**Figure 3E**).

Regarding to the autophagy-related proteins, overexpressing YAP increased the expression of LC3-II/I, Beclin-1, ATG5, and ATG7 and decreased the expression of p62, while knocking down YAP had the opposite effect (**Figure 4A**). The AD+sh-YAP+RPM group had partially increased LC3-II/I, Beclin-1, ATG5, and ATG7 and mildly decreased p62 expression. Overexpression of YAP with AD increased the number of LC3 fluorescence points, and the number of fluorescence points was localized with YAP and LC3. Knocking down YAP expression decreased the number of LC3 fluorescence points and the number of fluorescence points co-localized with YAP and LC3. After knocking down YAP and applying rapamycin, the number of LC3 and fluorescence points co-localized with YAP and LC3 increased again (**Figures 4B-D**, **Supplementary Figure S1**). It should be noted that overexpression of YAP alone can also cause an increase in LC3 fluorescence points and co-localization of YAP and LC3 (*P* < 0.05 compared to the Ctrl group and BLK group) while knocking down YAP alone can also cause a decrease in LC3 fluorescence points and co-localization of YAP and LC3 (*P* < 0.05 compared to the Ctrl group and sh-NC group). Transmission electron microscopy showed a significant decrease in autophagosome count in the AD cell model, which increased after overexpression of YAP and decreased with knockdown, then recovered slightly with rapamycin (**Figure 4E**). It should be noted that overexpression of YAP alone can also lead to an increase in the number of autophagosomes (*P* < 0.05 compared to the Ctrl group and the BLK group) and simply knocking down YAP can also decrease in the number of autophagosomes (*P* < 0.05 compared to the Ctrl group and the sh-NC group). The above results suggest that the decreased autophagy function in AD is likely related to the decreased expression of YAP.

### 3.5 YAP expression levels also affect the proliferation and apoptosis of AD-like inflammatory cells

**Figure 5:**
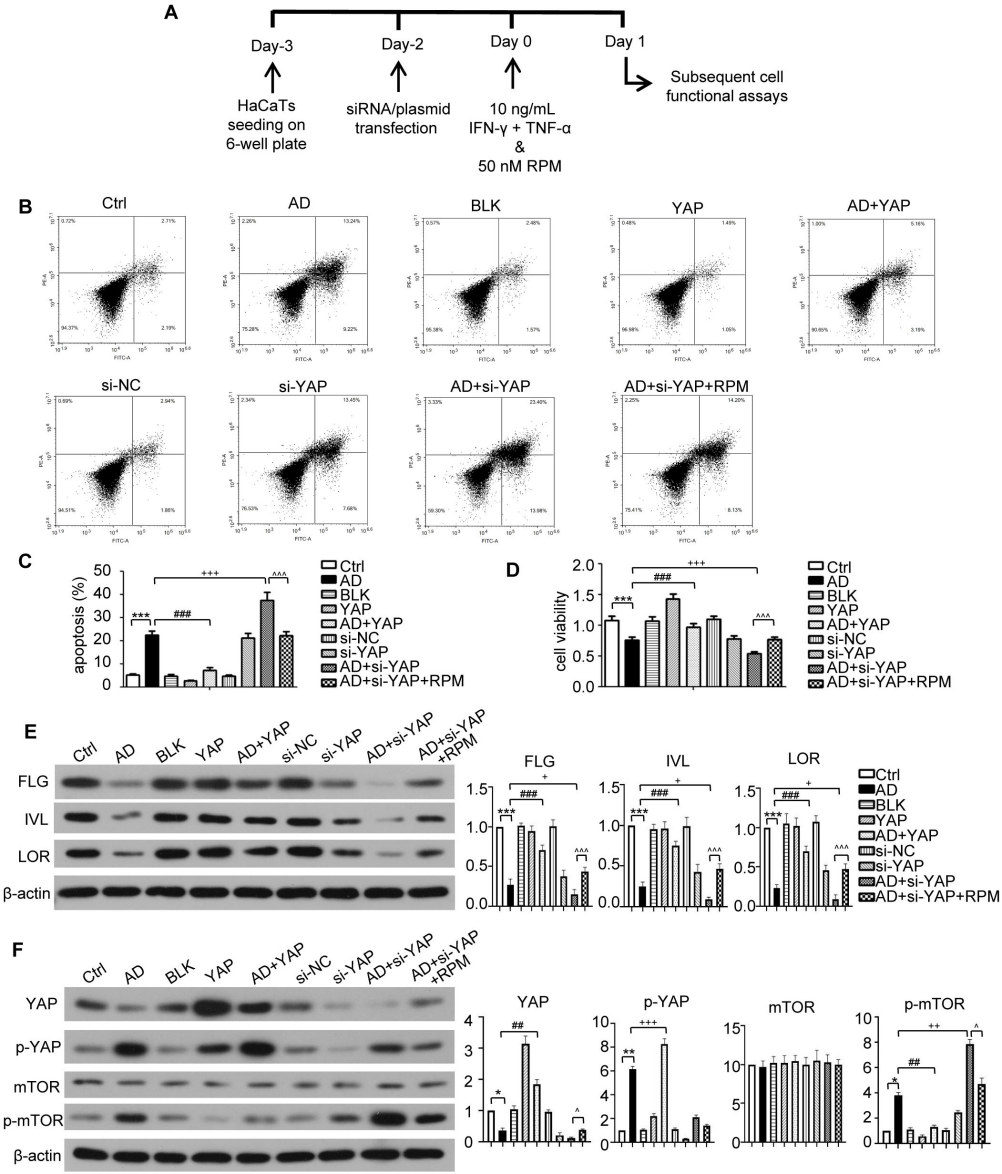
The effect of YAP expression on skin barrier in AD like inflammatory cell model. **(A)** The schematic overview of atopic dermatitis (AD) like inflammatory cell model. **(B)** Flow cytometry analysis of cell apoptosis rate results. **(C)** Cell apoptosis from flow cytometry analysis. **(D)** Cell viability from MTT analysis. **(E)** FLG, IVL, and LOR protein expression from western blot with the quantification results presented in the graphs. **(F)** YAP, p-YAP, mTOR, and p-mTOR protein expression from western blot with the quantification results presented in the graphs. Ctrl, control cells; AD, atopic dermatitis-like inflammatory cells; BLK, cells transfected with empty vector; YAP, cells transfected with YAP overexpression plasmid; AD+YAP, atopic dermatitis model cells transfected YAP overexpression plasmid; si-NC, cells transfected with si-NC; si-YAP, cells transfected with si-YAP; AD+si-YAP, atopic dermatitis model cells transfected with si-YAP; AD+si-YAP+RPM, AD+si-YAP group with 50 nM rapamycin co-culture. *P < 0.05, **P < 0.01, ***P < 0.001 Ctrl v.s. AD; ##*P* < 0.01, ###*P* < 0.001 AD+YAP v.s. AD; +*P* < 0.05, ++*P* < 0.01, +++*P* < 0.001 AD+si-YAP v.s. AD; ^*P* < 0.05, ^^^*P* < 0.001 AD+si-YAP+RPM v.s. AD+si-YAP.

*In vitro* experiments in an AD inflammatory cell model were next used to investigate the mechanisms in more detail (**Figure 5A**). The flow cytometry results are displayed in **Figure 5B**. The apoptosis level of AD-like inflammatory cells increased, but overexpression of YAP decreased apoptosis. Conversely, YAP knockdown increased apoptosis in AD-like inflammatory cells (**Figure 5C**). The proliferation ability of AD-like inflammatory cells was decreased compared to controls, but overexpression of YAP increased their proliferation while knocking down YAP decreased their proliferation, then adding rapamycin restored their proliferation ability (**Figure 5D**).

### 3.6 YAP influences barrier-related proteins and autophagy function of AD-like inflammatory cells

**Figure 6:**
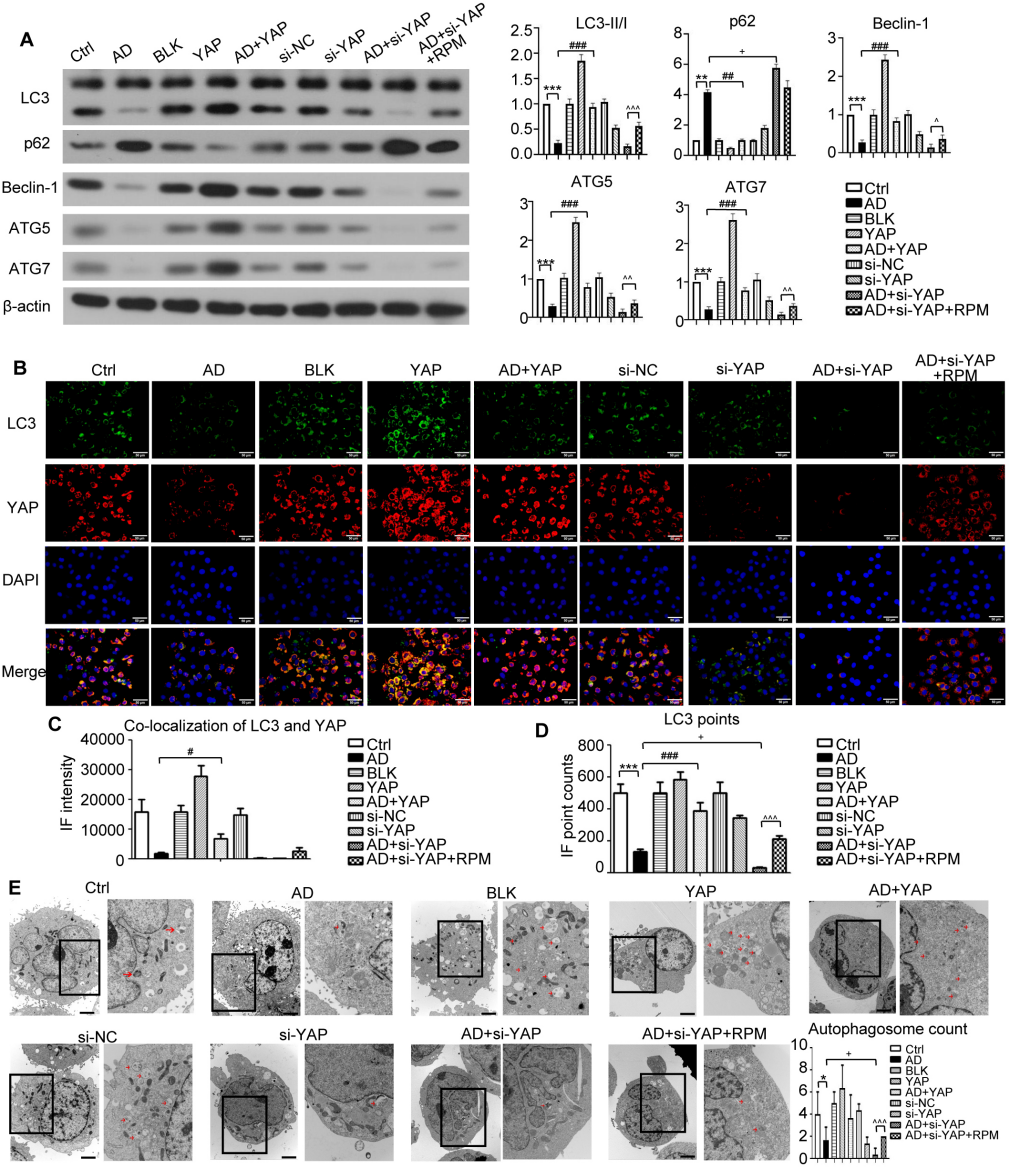
The effect of YAP expression on autophagy in the AD like inflammatory cell model. **(A)** Beclin-1, ATG5, ATG7, LC3, and p62 protein expression from western blot with quantification results presented in the graphs. **(B)** LC3 positive cells (×600) and LC3-YAP co-localization (×400) from immunofluorescence, bar length = 50 μm. LC3: green. YAP: red. **(C, D)** Graphs showing co-localization of LC3 and YAP, and the LC3 points. **(E)** Electron microscopy results and autophagosome count (left: ×10,000; right: ×20,000; bar length = 2 μm). Ctrl, control cells; AD: atopic dermatitis-like inflammatory cells; BLK, cells transfected with empty vector; YAP, cells transfected with YAP overexpression plasmid; AD+YAP, atopic dermatitis model cells transfected YAP overexpression plasmid; si-NC, cells transfected with si-NC; si-YAP, cells transfected with si-YAP; AD+si-YAP, atopic dermatitis model cells transfected with si-YAP; AD+si-YAP+RPM, AD+si-YAP group with 50 nM rapamycin co-culture. *P < 0.05, **P < 0.01, ***P < 0.001 Ctrl v.s. AD; #*P* < 0.05, ###*P* < 0.001 AD+YAP v.s. AD; +*P* < 0.05 AD+si-YAP v.s. AD; ^*P* < 0.05, ^^*P* < 0.01, ^^^*P* < 0.001 AD+si-YAP+RPM v.s. AD+si-YAP.

Western blots of FLG, IVL, and LOR in AD-like inflammatory cells demonstrated that overexpression of YAP increased their expression levels, knocking down YAP expression reduced their expression levels, but this was partially restored by adding rapamycin (**Figure 5E**). While the levels of mTOR were largely unchanged, the trend of changes in p-mTOR (**Figure 5F**), which reflects the activity of the mTOR signaling pathway, was similar to that of p62 (**Figure 6A**). In order to investigate this in more detail the levels of p-mTOR and mTOR at different time points in AD-like inflammatory cells were examined. The results showed that p-mTOR levels gradually decreased over time after YAP overexpression, and gradually increased over time after YAP knockdown (**Supplementary Figure S2**). Compared with the AD modeling group, overexpression of YAP increased the expression of LC3-II/I and decreased the expression of p62 in AD-like inflammatory cells. An opposite effect was found from knocking down YAP expression, and this was partially reversed by adding rapamycin (**Figure 6A**).

The immunofluorescence experiment showed that AD-like inflammatory cells overexpressing YAP had an increase in LC3 fluorescence points and an increase in YAP and LC3 co-localization fluorescence points. In contrast, knocking down YAP expression decreased the LC3 fluorescence points and decreased YAP and LC3 co-localization fluorescence points, and it was restored to a degree by rapamycin (**Figures 6B-D**, **Supplementary Figure S3**). It should be noted that overexpression of YAP alone also caused an increase in LC3 fluorescence points (*P* < 0.05 compared to the Ctrl group and the BLK group). Although YAP and LC3 co-localization also increased, the difference did not reach statistical significance. Simply knocking down YAP can also cause a decrease in the number of LC3 fluorescence points (*P* < 0.05 compared to the Ctrl group in the si-YAP group and the si-NC group). Although YAP and LC3 co-localization decreased, the difference did not reach statistical significance.

Transmission electron microscopy showed a significant decrease in autophagosome count in the AD cell model, which was increased by YAP overexpression and decreased by knocking down YAP. Knocking down YAP and treatment with rapamycin resulted in an increase in autophagosome count (**Figure 6E**). It should be noted that overexpression of YAP alone also caused an increase in the number of autophagosomes (*P* < 0.05 compared to the Ctrl group and *P* < 0.05 compared to the BLK group), and knockdown of YAP alone also caused a decrease in the number of autophagosomes (*P* < 0.05 compared to the Ctrl group and compared to the si-NC group). The above results are consistent with the investigations in the mouse model, indicating that increasing YAP expression can play a certain role in autophagy recovery in AD.

## 4 Discussion

This study explored whether downregulated YAP in AD reduces autophagy and if it influences skin barrier function. The results showed that YAP was expressed at a low level in patients’ AD samples and correlated with FLG expression. In a mouse AD model, YAP overexpression decreased the severity of the skin lesions alongside decreases in inflammation and increased barrier function. YAP overexpression increased LC3-II/I expression and decreased p62 and p-mTOR, while LC3 fluorescence points were increased alongside the number of fluorescence points co-localized with YAP, suggesting YAP-regulated autophagy. Meanwhile, YAP knockdown increased skin lesion severity, increased inflammation, decreased barrier function, and apparently decreased autophagy. However, adding rapamycin to the lesions reversed the effects of YAP knockdown to some degree. These results were supported by an investigation into AD-like inflammatory cells, which also showed that proliferation increased and apoptosis decreased. Again, YAP knockdown induced the opposite findings, but rapamycin reduced those effects. Overall, these results suggest that YAP might be a target for AD therapy, as increased expression levels seem to induce autophagy and improve inflammation and barrier function of AD.

The specific pathways involved in the mechanisms of atopic dermatitis are currently unclear. However, based on previous studies, YAP has an important role. This study demonstrates that the expression of YAP decreases in AD keratinocytes, and its expression level can affect the expression of skin barrier proteins. Increasing the expression of YAP can increase the expression of the skin barrier proteins FLG, IVL, and LOR. It is speculated that YAP may damage the barrier function of the epidermis by reducing the proliferation ability of keratinocytes, hindering differentiation and other effects, leading to clinical phenomena such as skin lesion erosion and difficult healing. In related studies in the field of skin, Schlegelmilch et al. (17) confirmed that YAP can regulate epidermal stem cell proliferation and maintain the three-dimensional structure of the skin by interacting with the transcription factor TEAD. Mice with YAP knockdown showed thinning of the epidermis, reduced stratum corneum, and disordered arrangement of the epidermal structure. Overexpression of YAP in normal human primary keratinocytes can promote immortalized cell proliferation, hinder their normal differentiation process, increase the marker molecules p63 and PCNA for epithelial proliferation, and decrease the marker molecules 14-3-3σ and LEKTI for differentiation (35). After knocking down YAP, the expression of transforming growth factor (TGF)-β1 decreased, the proliferation rate of epidermal basal layer cells slowed down, and the healing process of skin wounds was inhibited, confirming that the healing process of skin wounds depends on the expression of YAP (36).

This study hypothesized that downregulated YAP in AD influences autophagy. A reduced level of autophagy is involved in the core pathogenesis of skin barrier dysfunction in AD. It can regulate inflammatory responses, keratinocyte differentiation, and host defense against pathogens such as *Staphylococcus aureus*. When autophagy is inhibited, epidermal barrier function is reduced (13). The expression of YAP is crucial for maintaining cellular autophagy (37). Autophagy can also promote cell proliferation by activating YAP nuclear localization (37). According to the results of this study, the overexpression of YAP in normal mouse skin and keratinocytes can increase autophagy levels. At the same time, the knockdown of YAP can reduce autophagy levels. It was confirmed by changes in the autophagy-related proteins LC3, p62, and p-mTOR (38, 39). This study also confirms that reducing autophagy levels and increasing YAP expression in AD can promote keratinocyte proliferation, inhibit apoptosis, inhibit the mTOR signaling pathway activity, and partially restore cell autophagy function. These results suggest that the reduction of YAP in AD epidermal keratinocytes can inhibit the autophagy pathway through the mTOR signaling pathway and thus participate in the core mechanism of skin barrier dysfunction.

**Figure 7:**
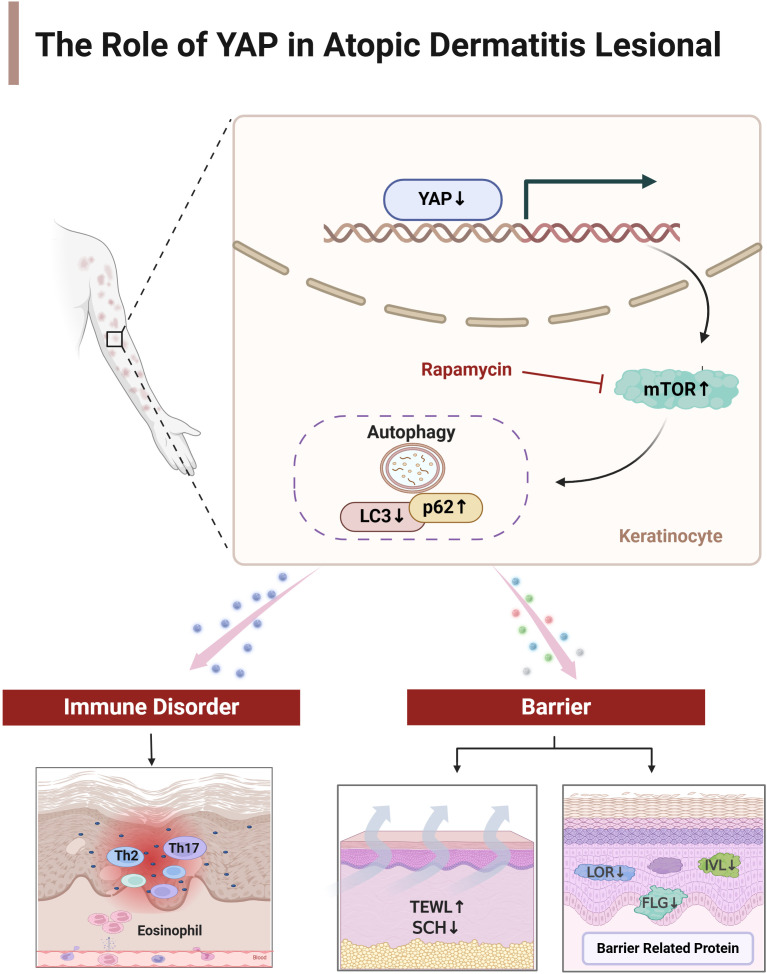
The mechanism of YAP on immune balance disorder and skin barrier dysfunction in atopic dermatitis. Created by Biorender.com

Based on previous research findings by the authors, YAP may be a key link in causing Th17/Treg imbalance in AD. Inhibiting YAP expression reduces the proportion of initial CD4+T cells differentiating into Th17 and Treg cells while overexpression increases (16). It suggests that changes in YAP expression, a key factor, play an important role in the two major mechanisms of AD immune balance disorder and skin barrier dysfunction (**Figure 7**). Targeted AD treatment may achieve a dual effect of regulating AD immune balance disorder and restoring the skin barrier. However, the expression trend of YAP in T cell subsets and skin keratinocytes is different, and further in-depth research and drug development are still a long process.

This study has some limitations. As a study that included only a few clinical samples and was largely based on animals and cultured cells, further study is needed to establish whether these changes in autophagy, inflammation, and skin barrier function translate into the clinical situation in humans. A more detailed investigation of the mechanisms should help fill in the details of how YAP regulates autophagy.

In conclusion, a decrease in YAP levels in AD leads to a decrease in autophagy levels and skin barrier dysfunction in AD. Therefore, enhancing the expression of YAP, inhibiting the mTOR pathway, and inducing autophagy may be considered new targets for treating AD.

## Data Availability

The original contributions presented in the study are included in the article/**Supplementary Material**. Further inquiries can be directed to the corresponding author.
