# Supplementary material for: Yes-associated protein regulates autophagy to restore skin barrier function in atopic dermatitis
Source: Front Immunol. 2025 Nov 19;16:1681148. doi: 10.3389/fimmu.2025.1681148 (PMC12672505; doi:10.3389/fimmu.2025.1681148)
Supplement: Supplementary file 1 [file Table1.docx]

**Supplementary Table S1. siRNAs and shRNAs sequences used in this study**

| Name | Sequences |
| --- | --- |
| si-YAP/shYAP | F：5’-CUGCCACCAAGCUAGAUAATT-3’  R：5’-UUAUCUAGCUUGGUGGCAGTT-3’ |
| Negative control | F：5’-GCGACGAUCUGCCUAAGAUTT-3’  R：5’-AUCUUAGGCAGAUCGUCGCTT-3’ |

**Supplementary Table S2. The dermatitis score and ear thickness of the mice of different sexes**

| Group | dermatitis score | | ear thickness (mm) | |
| --- | --- | --- | --- | --- |
|  | male | female | male | female |
| Ctrl | 0 | 0 | 0.140±0.010 | 0.153±0.015 |
| AD | 3.667±1.528 | 3.667±0.577 | 0.190±0.010 | 0.193±0.023 |
| BLK | 0 | 0 | 0.153±0.015 | 0.147±0.021 |
| YAP | 0 | 0 | 0.177±0.006 | 0.170±0.010 |
| AD+YAP | 2.000±1.000 | 2.000±1.000 | 0.180±0.020 | 0.177±0.025 |
| sh-NC | 0 | 0 | 0.143±0.006 | 0.153±0.021 |
| sh-YAP | 0 | 0 | 0.157±0.015 | 0.153±0.015 |
| AD+sh-YAP | 6.667±1.528 | 6.333±1.528 | 0.257±0.015 | 0.273±0.029 |
| AD+sh-YAP+RPM | 4.333±1.155 | 4.333±2.082 | 0.227±0.015 | 0.223±0.006 |

**Supplementary Table S3. Trans Epidermal Water Loss (TEWL) of the mice of different sexes (g/m^2^/h)**

| Group | Week 1 | | Week 2 | | Week 3 | |
| --- | --- | --- | --- | --- | --- | --- |
|  | male | female | male | female | male | female |
| Ctrl | 6.222±0.509 | 6.222±0.509 | 6.444±0.770 | 6.889±0.385 | 6.778±0.839 | 6.555±0.385 |
| AD | 6.889±1.071 | 7.667±1.764 | 10.444±0.839 | 11.445±1.678 | 17.333±1.764 | 16.333±2.000 |
| BLK | 6.667±1.000 | 6.889±1.644 | 6.778±0.839 | 5.778±0.694 | 7.333±1.333 | 5.667±0.882 |
| YAP | 6.222±0.770 | 6.889±1.071 | 6.889±0.839 | 5.222±1.262 | 6.111±1.347 | 7.889±1.018 |
| AD+YAP | 6.778±1.018 | 7.778±0.509 | 8.556±0.193 | 8.778±0.839 | 13.889±0.385 | 12.667±2.082 |
| sh-NC | 6.222±0.509 | 7.222±0.770 | 7.000±1.000 | 5.778±0.509 | 6.333±0.667 | 6.445±0.385 |
| sh-YAP | 5.778±0.962 | 7.444±1.171 | 7.556±0.839 | 6.556±0.509 | 6.222±0.192 | 5.889±1.071 |
| AD+sh-YAP | 7.111±0.509 | 8.667±0.334 | 15.000±0.667 | 14.111±1.388 | 24.667±3.180 | 23.555±1.347 |
| AD+sh-YAP+RPM | 7.444±1.836 | 7.667±1.000 | 11.222±1.347 | 11.889±0.694 | 19.333±1.333 | 21.000±0.882 |

**Supplementary Table S4. Stratum corneum hydration (SCH) of the mice of different sexes (a.u.)**

| Group | Week 1 | | | Week 2 | | | Week 3 | | |
| --- | --- | --- | --- | --- | --- | --- | --- | --- | --- |
|  | male | female | | male | female | | male | female | |
| Ctrl | 20.333±1.764 | | 21.000±0.333 | 20.666±0.577 | | 22.111±1.710 | 20.667±0.334 | | 19.111±0.385 |
| AD | 19.222±1.347 | | 18.000±1.155 | 14.778±1.018 | | 14.889±0.192 | 11.222±0.839 | | 10.000±0.577 |
| BLK | 19.334±0.577 | | 21.111±1.575 | 23.111±0.839 | | 22.667±1.764 | 19.667±2.906 | | 21.778±1.644 |
| YAP | 18.889±0.509 | | 20.667±1.453 | 22.222±1.895 | | 20.667±0.667 | 20.889±1.896 | | 20.111±3.339 |
| AD+YAP | 18.778±1.503 | | 17.555±2.117 | 15.889±1.503 | | 17.111±0.962 | 13.444±0.509 | | 12.667±0.882 |
| sh-NC | 20.445±2.037 | | 20.444±1.171 | 20.556±3.271 | | 22.111±1.018 | 22.333±0.334 | | 20.445±2.546 |
| sh-YAP | 19.556±1.262 | | 20.445±0.385 | 21.444±0.962 | | 22.778±0.509 | 21.000±3.606 | | 22.777±1.347 |
| AD+sh-YAP | 16.889±1.170 | | 16.111±1.710 | 10.000±0.882 | | 11.111±3.151 | 3.222±1.388 | | 2.555±1.072 |
| AD+sh-YAP+RPM | 17.111±1.836 | | 17.778±1.018 | 13.555±1.347 | | 10.222±1.387 | 5.778±1.072 | | 6.667±1.000 |


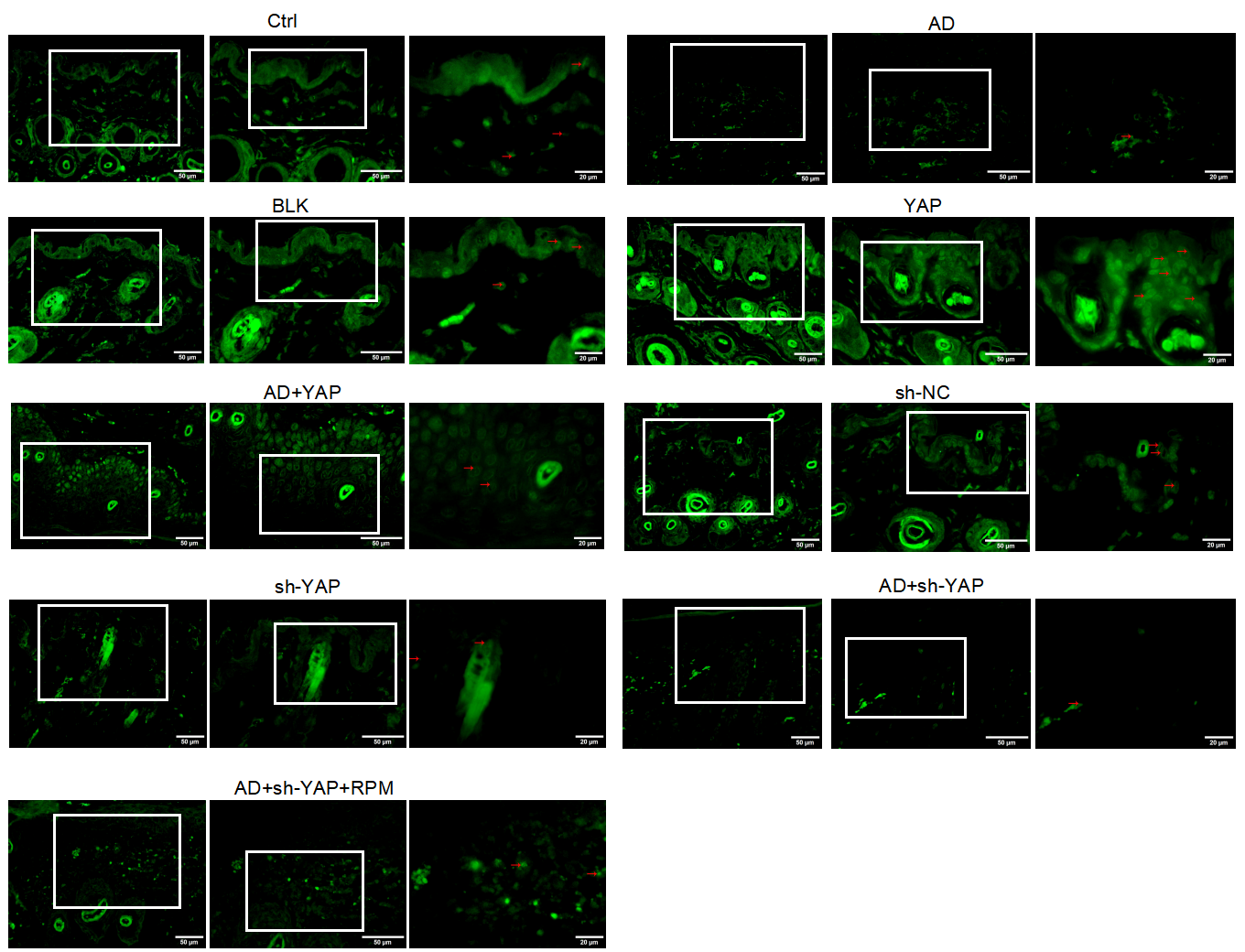


**Supplementary Figure S1. LC3 immunofluorescence at varying magnifications in AD mouse models.**

left: 400×; middle: 600×; right:1000×.

Ctrl: control mice; AD: atopic dermatitis model mice; BLK: mice injected with empty vector lentivirus; YAP: mice injected with YAP overexpression lentivirus; AD+YAP: atopic dermatitis model mice injected with YAP overexpression lentivirus; sh-NC: mice injected with shRNA-NC lentivirus; sh-YAP: mice injected with YAP shRNA lentivirus; AD+sh-YAP: atopic dermatitis model mice injected with YAP shRNA lentivirus; AD+sh-YAP+RPM: AD+sh-YAP group with topical 0.2% rapamycin ointment.

**
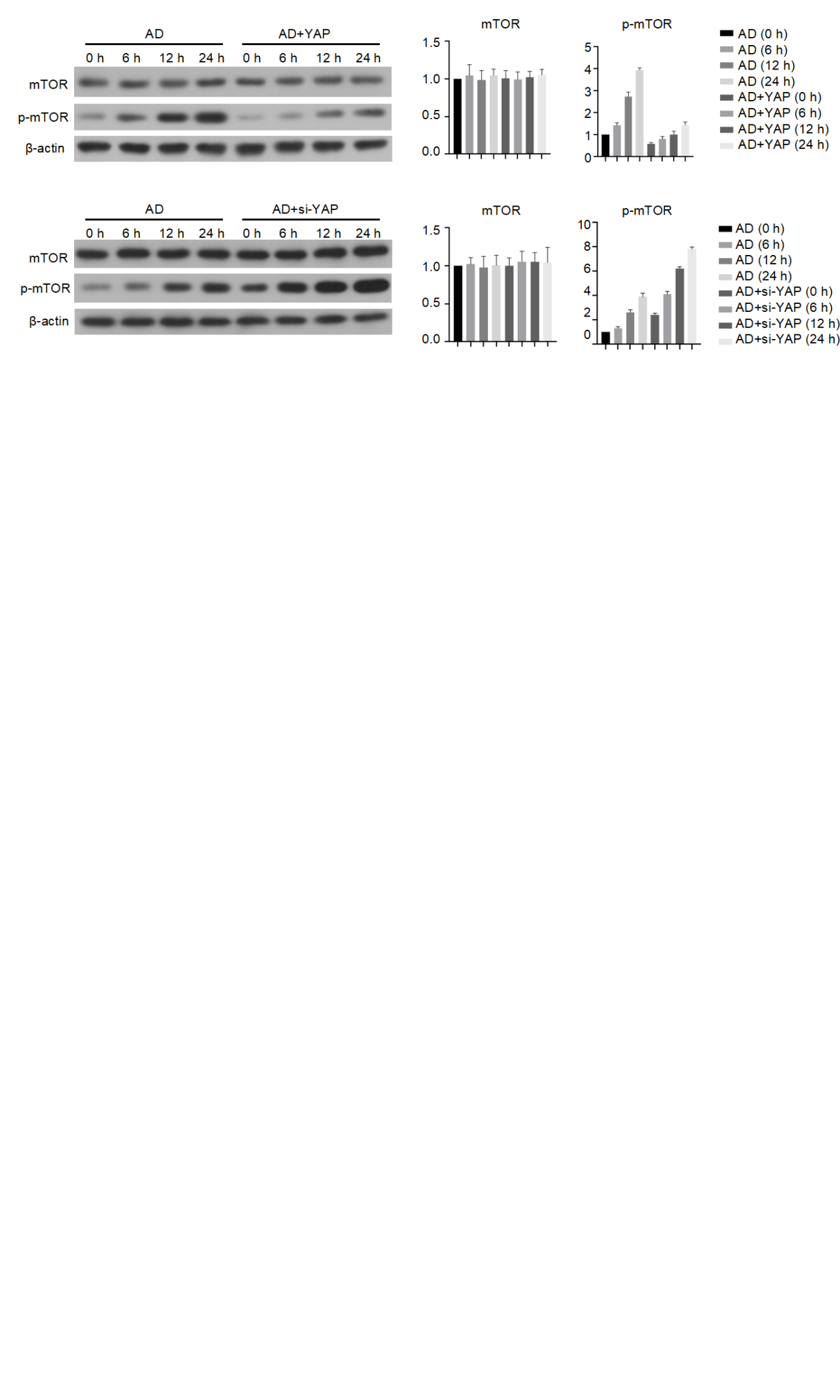
Supplementary Figure S2. The effect of YAP expression on mTOR and p-mTOR at varying times in an AD-like inflammatory cell model.**

AD: atopic dermatitis-like inflammatory cells; AD+YAP: atopic dermatitis model cells transfected YAP overexpression plasmid; AD+si-YAP: atopic dermatitis model cells transfected with si-YAP.


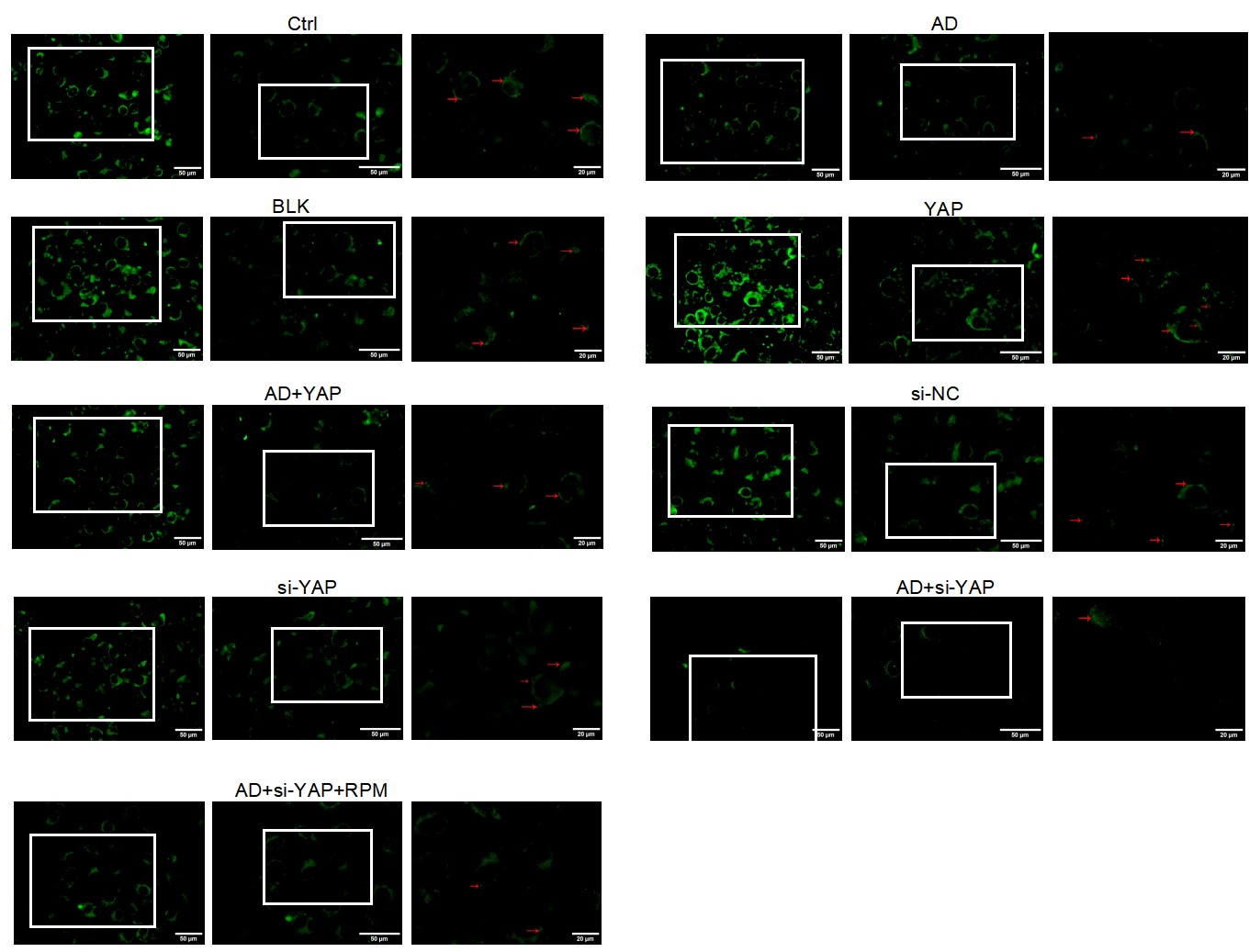


**Supplementary Figure S3. LC3 immunofluorescence at varying magnifications in an AD-like inflammatory cell model.**

left: 400×; middle: 600×; right:1000×.

Ctrl: control cells; AD: atopic dermatitis like inflammatory cells; BLK: cells transfected with empty vector; YAP: cells transfected with YAP overexpression plasmid; AD+YAP: atopic dermatitis model cells transfected YAP overexpression plasmid; si-NC: cells transfected with si-NC; si-YAP: cells transfected with si-YAP; AD+si-YAP: atopic dermatitis model cells transfected with si-YAP; AD+si-YAP+RPM: AD+si-YAP group with 50 nM rapamycin co-culture.
